# Differences in Colorectal Cancer Awareness Between Vegetarians and Nonvegetarians: A National Cross-Sectional Study From Palestine

**DOI:** 10.1200/GO.23.00400

**Published:** 2024-02-22

**Authors:** Mohamedraed Elshami, Maram Albandak, Mohammed Alser, Ibrahim Al-Slaibi, Mohammed Ayyad, Mohammad F. Dwikat, Shoruq A. Naji, Balqees M. Mohamad, Wejdan S. Isleem, Adela Shurrab, Bashar Yaghi, Yahya Ayyash Qabaja, Fatma K. Hamdan, Raneen R. Sweity, Remah T. Jneed, Khayria A. Assaf, Mohammed M. Hmaid, Iyas I. Awwad, Belal K. Alhabil, Marah N. Alarda, Amani S. Alsattari, Moumen S. Aboyousef, Omar A. Aljbour, Rinad AlSharif, Christy T. Giacaman, Ali Y. Alnaga, Ranin M. Abu Nemer, Nada M. Almadhoun, Sondos M. Skaik, Shurouq I. Albarqi, Nasser Abu-El-Noor, Bettina Bottcher

**Affiliations:** ^1^ Division of Surgical Oncology, Department of Surgery, University Hospitals Cleveland Medical Center, Cleveland, OH; ^2^ Ministry of Health, Gaza, Palestine; ^3^ Faculty of Medicine, Al-Quds University, Jerusalem, Palestine; ^4^ The United Nations Relief and Works Agency for Palestine Refugees in the Near East (UNRWA), Gaza, Palestine; ^5^ Almakassed Hospital, Jerusalem, Palestine; ^6^ Faculty of Medicine, An-Najah National University, Nablus, Palestine; ^7^ Faculty of Pharmacy, Al-Azhar University of Gaza, Gaza, Palestine; ^8^ Doctors Without Borders (Médecins Sans Frontières), Hebron, Palestine; ^9^ Faculty of Medicine, Islamic University of Gaza, Gaza, Palestine; ^10^ Palestine Medical Complex, Khanyounis, Palestine; ^11^ Faculty of Dentistry, Arab American University, Jenin, Palestine; ^12^ Augusta Victoria Hospital, Jerusalem, Palestine; ^13^ Faculty of Allied Medical Sciences, Arab American University, Jenin, Palestine; ^14^ Faculty of Medicine, Al-Azhar University, Gaza, Palestine; ^15^ Faculty of Nursing, Islamic University of Gaza, Gaza, Palestine

## Abstract

**PURPOSE:**

To compare colorectal cancer (CRC) awareness between vegetarians and nonvegetarians in Palestine.

**MATERIALS AND METHODS:**

The validated Bowel Cancer Awareness Measure and Cancer Awareness Measure-Mythical Causes Scale were translated into Arabic and used to assess awareness of CRC signs/symptoms, risk factors, and mythical causes. The total awareness score of each domain was calculated and categorized into tertiles; the top tertile was considered as good awareness. Multivariable logistic regression analysis was used to examine the association between being a vegetarian and displaying good awareness in each domain.

**RESULTS:**

This study included 4,623 participants: 560 vegetarians (12.1%) and 4,063 nonvegetarians (87.9%). Lump in the abdomen was the most recognized CRC sign/symptom among both nonvegetarians (n = 2,969, 73.1%) and vegetarians (n = 452, 80.7%). Vegetarians were less likely than nonvegetarians to display good awareness of CRC signs/symptoms (odds ratio, 0.59 [95% CI, 0.48 to 0.72]). Lack of physical activity was the most identified modifiable CRC risk factor in both nonvegetarians (n = 3,368, 82.9%) and vegetarians (n = 478, 85.4%). Similarly, having a bowel disease was the most identified nonmodifiable risk factor among both nonvegetarians (n = 2,889, 71.1%) and vegetarians (n = 431, 77.0%). There were no associated differences between both groups in the awareness levels of CRC risk factors. The most recognized food-related CRC causation myth in nonvegetarians was drinking from plastic bottles (n = 1,023, 25.2%), whereas it was eating burnt food in vegetarians (n = 176, 31.4%). Having a physical trauma was the most recognized food-unrelated myth in both nonvegetarians (n = 2,356, 58.0%) and vegetarians (n = 396, 70.7%). There were no associated differences in the awareness of CRC causation myths between both groups.

**CONCLUSION:**

Awareness of CRC was notably low in both Palestinian vegetarians and nonvegetarians. Particularly, vegetarians demonstrated lower awareness of CRC signs and symptoms.

## Teaser

A study from Palestine showed differences in colorectal cancer awareness between vegetarians and nonvegetarians.

## INTRODUCTION

In Palestine, colorectal cancer (CRC) ranks second after breast cancer, with an incidence rate of 15.3 and 10.2 per 100,000 general population in the West Bank and the Gaza Strip, respectively.^1^ Obesity, high consumption of processed red meat, low-fiber and limited intake of fruits and vegetables, and drinking alcohol are all recognized as significant diet-related contributors to the increasing incidence of CRC.^2^ Hence, dietary factors continue to represent a significant portion of CRC modifiable risk factors, and the pursuit of dietary interventions for primary prevention of CRC remains a desirable objective.

**CONTEXT**

**Key Objective**
In Palestine, colorectal cancer (CRC) remains a major public health concern with significant morbidity and mortality. Diet is a significant modifiable risk factor for CRC, and plant-based diets, such as vegetarianism, may potentially influence CRC awareness and health behaviors. Therefore, this study explored the differences in CRC awareness levels between vegetarians and nonvegetarians in Palestine.
**Knowledge Generated**
Awareness of CRC signs/symptoms, risk factors, and causation myths was notably low in both Palestinian vegetarians and nonvegetarians. Particularly, vegetarians demonstrated lower awareness of CRC signs and symptoms.
**Relevance**
In-person and virtual awareness campaigns are needed to address the knowledge gaps observed among vegetarians, specifically to improve the low awareness of CRC signs and symptoms within this group. These initiatives may pave the way for the establishment of a CRC screening program in Palestine, ultimately contributing to the early detection of CRC and fostering improved health outcomes.

Plant-based diets, including both vegan and vegetarian diets, are increasingly becoming more prevalent. On a global scale, the market for dairy product alternatives is projected to attain a value of $25 billion (US dollars) by 2026, indicating a growing consumer preference for nonanimal products.^3^ Adherence to vegetarian dietary patterns has been linked to several favorable health outcomes, including decreased prevalence of obesity, metabolic syndrome, type 2 diabetes mellitus, and hypertension, as evidenced in the Adventist Health Study-2.^4^ Moreover, vegetarian diets have been linked to an overall decreased risk of colon cancer.^4^ Thus, dietary factors can contribute to an increased risk or exert a protective effect in the development of CRC.^2^ Should these associations prove to be causal, they could hold significant implications for the primary prevention of CRC.^4^

In light of the escalating global incidence of CRC, particularly in developing nations that are increasingly embracing more western dietary and lifestyle habits, more studies are warranted to further explore the influence of different dietary trends on the awareness and attitudes concerning CRC. In this national study, we aimed to evaluate the awareness levels of CRC signs/symptoms, risk factors, and causation myths among vegetarians compared with nonvegetarians in Palestine. We also examined the association between following a vegetarian diet and exhibiting good awareness of CRC.

## MATERIALS AND METHODS

### Study Design and Population

**FIG 1:**
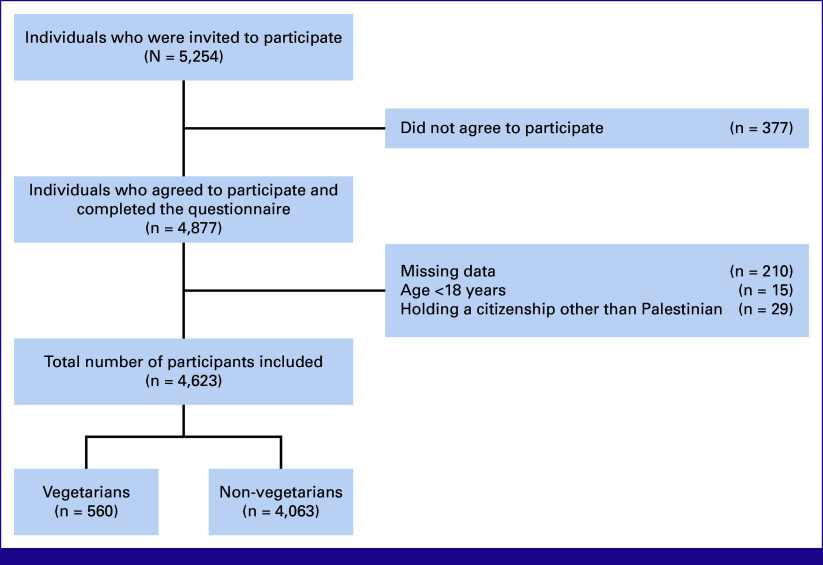
CONSORT diagram for selecting the study participants.

This was a nationwide cross-sectional study executed between July 16, 2019, and March 31, 2020. The target population was Palestinian adults 18 years and older residing in the West Bank, Jerusalem, and the Gaza Strip, comprising about 62.2% of about 5 million Palestinians.^5^ The inclusion criteria comprised being a Palestinian adult and visiting one of the designated data collection sites. Conversely, the exclusion criteria encompassed individuals visiting oncology departments at the time of data collection, studying, or working in the medical field, possessing citizenship other than Palestinian, and being unable to complete the questionnaire. Figure 1 summarizes the selection of the study participants.

### Sampling Methods

In Palestine, governmental hospitals and primary health care centers typically offer health care services with low co-payments or free of charge, making them the most commonly used services by the majority of patients.^1^ Palestine comprises 16 governorates, with five located in the Gaza Strip and 11 in the West Bank and Jerusalem.^6^ Consequently, convenience sampling was used to recruit participants from government hospitals, primary health care centers, and public spaces across 11 governorates in Palestine (four in the Gaza Strip and seven in the West Bank and Jerusalem) consistent with previous studies.^7-9^ Public spaces encompassed markets, places of worship (churches and mosques), malls, parks, downtown areas, and public transportation stations.

### Data Collection and Measurement Tool

For this study, two assessment tools were used to collect data on public awareness of CRC signs/symptoms, risk factors, and causation myths: the Bowel Cancer Awareness Measure (BoCAM) and the Cancer Awareness Measure-Mythical Causes Scale (CAM-MYCS). Both are validated questionnaires developed by University College London and Cancer Research, UK.^10,11^ A modified version of the BoCAM, and the CAM-MYCS, after back-to-back translation, was used for data collection. The questionnaire was translated from English to Arabic by two bilingual health care professionals and then independently back-translated to English by two other health care professionals. All those health care professionals were equally proficient in both languages with experience in clinical research and survey design. In addition, the questionnaire underwent subsequent review by five independent experts in the fields of public health, gastroenterology, and coloproctology to further ensure content validity and accuracy of translation. To assess the clarity of the questions of the Arabic version of the questionnaire, a pilot study was performed (n = 25). The questionnaires of the pilot study were not included in the final analysis. Finally, Cronbach's alpha was used to evaluate the internal consistency of the questionnaire and it yielded an acceptable value of .87.

The questionnaire consisted of four sections. The first section described sociodemographic factors of study participants. The second section assessed the participants' awareness of 12 CRC signs/symptoms, using a five-point Likert scale (1 = strongly disagree, 2 = disagree, 3 = not sure, 4 = agree, and 5 = strongly agree). The third section evaluated the participants' awareness of 11 CRC risk factors using the same five-point Likert scale. While nine of the 12 CRC signs/symptoms were retained from the original BoCAM,^10^ three additional symptoms, namely, feeling persistently full, unexplained loss of appetite, and unexplained generalized fatigue, were added to the questionnaire, on the basis of other versions of the Cancer Awareness Measure.^12,13^ Of the 11 CRC risk factors, 10 were adapted from the original BoCAM.^10^ Smoking cigarettes was added as it was deemed important given its high prevalence in Palestine.^14^ The questions from the original BoCAM, designed with yes/no/unknown responses, were adapted to the five-point Likert scale to reduce the likelihood of arbitrary responses.^7,15,16^ The fourth section assessed the participants' recognition of 13 myths around CRC causation to be incorrect. Of those 13 myths, 12 were adapted from the original CAM-MYCS.^11^ Eating burnt food was included as the 13th myth because of its prevalence as a belief within the Palestinian community.^17^

Data collectors used Kobo Toolbox (Cambridge, MA) to record the responses of study participants in face-to-face interviews. Kobo Toolbox is a secure and user-friendly data collection tool that is accessible via smartphones.^18^ Data collectors received comprehensive training to learn how to use Kobo Toolbox and to effectively guide participants in completing the questionnaire.

### Exposure and Outcomes

CRC awareness in vegetarians can influence lifestyle choices beyond dietary habits. Increased awareness may prompt vegetarian individuals to adopt overall healthier lifestyles.^19^ Previous studies from our group in Palestine found a suboptimal awareness of CRC in the general population.^7,8^ However, there is a paucity of studies examining CRC awareness in vegetarians and comparing it with that in nonvegetarians. Therefore, the exposure of interest in this follow-up study was following a vegetarian diet. Being a vegetarian was defined as self-identification by study participants to be on a diet characterized by the exclusion of meat, poultry, and seafood and to rely on plant-based foods for their nutritional needs. The primary outcome was the proportion of vegetarian participants displaying good awareness of CRC signs/symptoms, risk factors, and causation myths.

### Statistical Analysis

Continuous variables with non-normal distribution were summarized using median and IQR, and baseline comparisons between vegetarian and nonvegetarian participants were conducted using the Kruskal-Wallis test. Meanwhile, categorical variables were described using frequencies and percentages, and comparisons were performed using Pearson's chi-square test.

CRC signs/symptoms were categorized into three groups: those with mass or blood, those of nonspecific nature, and those with other GI signs/symptoms. Similarly, CRC risk factors were divided into modifiable and nonmodifiable categories. Prompt recognition of CRC signs/symptoms and risk factors was measured through questions on the basis of a five-point Likert scale, where strongly agree or agree represented correct responses, whereas strongly disagree, disagree, or not sure was considered incorrect answers. Myths surrounding the causation of CRC were categorized into two groups: food-related and food-unrelated. Answers with disagree or strongly disagree were considered correct, whereas all other responses were considered incorrect. Recognition of CRC signs/symptoms, risk factors, and causation myths was described using frequencies and percentages, with comparisons between vegetarian and nonvegetarian participants performed using Pearson's chi-square test. This was followed by performing multivariable logistic regression analysis to examine the association between being a vegetarian and recognizing each of the CRC signs/symptoms, risk factors, and causation myths. The multivariable model adjusted for age, sex, education, occupation, monthly income, place of residency, marital status, the presence of a chronic disease, knowing someone with cancer, and data collection site. This model was determined on the basis of previous research studies.^7-9,11,15,20-29^

The awareness level of each of the CRC signs/symptoms, risk factors, and causation myths was assessed using a scoring system that had also been used in previous studies.^7-9^ For each correctly recognized item, one point was given. The total awareness score of each domain was calculated and categorized into tertiles; the first tertile was considered poor awareness, the second tertile was considered fair awareness, and third tertile was considered good awareness. Awareness levels were described using frequencies and percentages, and comparisons were performed between vegetarian and nonvegetarian participants using Pearson’s chi-square test. This was followed by running multivariable logistic regression analysis to examine the association between being a vegetarian and displaying good awareness in each domain. The same aforementioned multivariable model was used.

Missing data were hypothesized to be missed completely at random, and thus, complete case analysis was used to handle them. Data were analyzed using Stata software version 17.0 (StataCorp, College Station, TX).

### Ethical Approval and Consent to Participate

Ethical approval had been sought and granted from the Research Ethics Committee at the Islamic University of Gaza, the Human Resources Development department at the Palestinian Ministry of Health, and the Helsinki Committee in the Gaza Strip before data collection. Participants were provided with a comprehensive overview of the study’s purpose and objectives with emphasis on the voluntary nature of their participation. Before questionnaire completion, written informed consent was obtained from each participant, and data were anonymously collected.

## RESULTS

### Participant Characteristics

This study included 4,623 participants. Of those, 560 (12.1%) identified themselves as vegetarians, whereas 4,063 (87.9%) reported being nonvegetarians. Nonvegetarian participants reported higher monthly income and more often knew someone with cancer (Table 1). Notably, a higher percentage of participants from the Gaza Strip (68.9%) reported following a vegetarian diet compared with those from the West Bank and Jerusalem (31.1%).

**TABLE 1:** Characteristics of Study Participants

Characteristic	Vegetarian (n = 560)	Nonvegetarian (n = 4,063)	*P*
Age, years, median (IQR)	31.5 (24-40)	31 (24-43)	.10
Age group, years, No. (%)			.023
18-44	458 (81.8)	3,150 (77.5)	
45 or older	102 (18.2)	913 (22.5)	
Male sex, No. (%)	118 (21.1)	1,761 (43.3)	<.001
Educational level, No. (%)			.30
Secondary or below	257 (45.9)	1,960 (48.2)	
Postsecondary	303 (54.1)	2,103 (51.8)	
Occupation, No. (%)			<.001
Unemployed/housewife	330 (58.9)	1,737 (42.8)	
Employed	143 (25.5)	1,755 (43.2)	
Retired	11 (2.0)	85 (2.1)	
Student	76 (13.6)	486 (12.0)	
Monthly income ≥1,450 NIS, No. (%)	229 (40.9)	2,810 (69.2)	<.001
Marital status, No. (%)			.054
Single	155 (27.7)	1,259 (31.0)	
Married	394 (70.4)	2,673 (65.8)	
Divorced/widowed	11 (2.0)	131 (3.2)	
Having a chronic disease, No. (%)	103 (18.4)	803 (19.8)	.44
Knowing someone with cancer, No. (%)	213 (38.0)	2,182 (53.7)	<.001
Place of residency, No. (%)			<.001
The Gaza Strip	386 (68.9)	1,537 (37.8)	
WBJ	174 (31.1)	2,526 (62.2)	
Site of data collection, No. (%)			<.001
Public spaces	218 (38.9)	1,232 (30.3)	
Hospitals	101 (18.0)	1,558 (38.3)	
Primary health care centers	241 (43.0)	1,273 (31.3)	

Abbreviations: NIS, New Israeli Shekel; WBJ, West Bank and Jerusalem.

### Recognition of CRC Signs/Symptoms

The most frequently recognized CRC sign/symptom among the nonvegetarian group was lump in the abdomen (n = 2,969, 73.1%), followed by unexplained weight loss (n = 2,934, 72.2%; Table 2). Similarly, lump in the abdomen was the most identified sign/symptom in the vegetarian group (n = 452, 80.7%), followed by blood in the stools (n = 389, 69.5%). Conversely, the least frequently recognized sign/symptom in the nonvegetarian group was pain in the back passage (n = 2,030, 50.0%), whereas in the vegetarian group, bowel does not completely empty after using the lavatory was the least identified (n = 191, 34.1%).

**TABLE 2:** Association Between Being a Vegetarian and Recognition of Colorectal Cancer Signs/Symptoms

Category of Sign/Symptom	Sign/Symptom	Vegetarian (n = 560), No. (%)	Nonvegetarian (n = 4,063), No. (%)	Adjusted OR^a^ (95% CI)	*P*
Signs/symptoms with a mass or blood	Lump in the abdomen	452 (80.7)	2,969 (73.1)	1.43 (1.13 to 1.80)	.003
	Blood in the stools	389 (69.5)	2,730 (67.2)	1.20 (0.98 to 1.47)	.08
	Bleeding from back passage	385 (68.8)	2,368 (58.3)	1.47 (1.21 to 1.80)	<.001
Signs/symptoms of a nonspecific nature	Unexplained weight loss	363 (64.8)	2,934 (72.2)	0.72 (0.59 to 0.87)	.001
	Unexplained generalized fatigue	316 (56.4)	2,909 (71.6)	0.53 (0.43 to 0.64)	<.001
	Unexplained loss of appetite	299 (53.4)	2,573 (63.3)	0.66 (0.55 to 0.80)	<.001
	Anemia	257 (45.9)	2,540 (62.5)	0.61 (0.50 to 0.73)	<.001
Other GI signs/symptom–	Feeling persistently full	221 (39.5)	2,523 (62.1)	0.42 (0.35 to 0.51)	<.001
	Change in bowel habits	214 (38.2)	2,472 (60.8)	0.45 (0.37 to 0.54)	<.001
	Persistent pain in the abdomen	285 (50.9)	2,389 (58.8)	0.85 (0.70 to 1.03)	.09
	Bowel does not completely empty after using the lavatory	191 (34.1)	2,213 (54.5)	0.50 (0.41 to 0.61)	<.001
	Pain in the back passage	192 (34.3)	2,030 (50.0)	0.57 (0.47 to 0.69)	<.001

Abbreviation: OR, odds ratio.

^a^ Adjusted for age group, sex, educational level, occupation, monthly income, place of residency, marital status, the presence of a chronic disease, knowing someone with cancer, and data collection site.

Vegetarians were less likely than nonvegetarians to recognize eight of 12 CRC signs/symptoms. They included all CRC sign/symptoms of nonspecific nature and all other GI signs/symptoms except persistent pain in the abdomen.

### Recognition of CRC Risk Factors

Lack of physical activity was the most identified modifiable risk factor for CRC among both nonvegetarian (n = 3,368, 82.9%) and vegetarian groups (n = 478, 85.4%; Table 3). By contrast, the modifiable risk factor with the least recognition in the nonvegetarian group was having a diet low in fiber (n = 1,696, 41.7%), whereas in the vegetarian group, it was consuming red meat once a day or more (n = 288, 51.4%).

**TABLE 3:** Association Between Being a Vegetarian and Recognition of Colorectal Cancer Risk Factors

Risk Factor	Vegetarian (n = 560), No. (%)	Nonvegetarian (n = 4,063), No. (%)	Adjusted OR^a^ (95% CI)	*P*
Modifiable risk factors				
Not doing 30 minutes of moderate physical activity five times a week	478 (85.4)	3,368 (82.9)	1.31 (1.01 to 1.71)	.041
Smoking cigarettes	443 (79.1)	3,035 (74.7)	1.03 (0.82 to 1.30)	.79
Drinking alcohol	416 (74.3)	3,002 (73.9)	0.77 (0.62 to 0.96)	.018
Not eating five portions of fruits and vegetables a day	443 (79.1)	2,831 (69.7)	1.65 (1.32 to 2.07)	<.001
Being overweight	309 (55.2)	2,796 (68.8)	0.59 (0.48 to 0.71)	<.001
Eating red meat once a day or more	288 (51.4)	2,180 (53.7)	0.82 (0.68 to 0.99)	.039
Having a diet low in fiber (eg, fruits and vegetables)	289 (51.6)	1,696 (41.7)	1.21 (1.00 to 1.46)	.051
Nonmodifiable risk factors				
Having a bowel disease (eg, inflammatory bowel disease)	431 (77.0)	2,889 (71.1)	1.23 (0.98 to 1.53)	.07
Having a close relative with bowel cancer	329 (58.8)	2,267 (55.8)	1.01 (0.84 to 1.23)	.89
Being over 70 years old	175 (31.3)	1,952 (48.0)	0.48 (0.39 to 0.58)	<.001
Having diabetes	207 (37.0)	1,374 (33.8)	0.94 (0.77 to 1.14)	.50

Abbreviation: OR, odds ratio.

^a^ Adjusted for age group, sex, educational level, occupation, monthly income, place of residency, marital status, the presence of a chronic disease, knowing someone with cancer, and data collection site.

Having a bowel disease was the most identified nonmodifiable risk factor among both nonvegetarians (n = 2,889, 71.1%) and vegetarians (n = 431, 77.0%). Conversely, the least recognized nonmodifiable risk factor in the nonvegetarian group was having diabetes (n = 1,374, 33.8%), and in the vegetarian group, it was being over 70 years old (n = 175, 31.3%).

Vegetarians were less likely than nonvegetarians to recognize four of 11 CRC risk factors. Those included drinking alcohol (odds ratio [OR], 0.77 [95% CI, 0.62 to 0.96]), being overweight (OR, 0.59 [95% CI, 0.48 to 0.71]), eating red meat once a day or more (OR, 0.82 [95% CI, 0.68 to 0.99]), and having diabetes (OR, 0.48 [95% CI, 0.39 to 0.58]).

### Recognition of CRC Causation Myths

In the assessment of public beliefs regarding the mythical causes of CRC, the most commonly reported food-related myth in the nonvegetarian group was drinking from plastic bottles (n = 1,023, 25.2%), whereas it was eating burnt food (n = 176, 31.4%) in the vegetarian group (Table 4). Conversely, eating food additives was the least reported food-related myth among both nonvegetarians (n = 372, 9.2%) and vegetarians (n = 84, 15.0%).

**TABLE 4:** Association Between Being a Vegetarian and Recognition of Colorectal Cancer Causation Myths

Myth	Vegetarian (n = 560), No. (%)	Nonvegetarian (n = 4,063), No. (%)	Adjusted OR^a^ (95% CI)	*P*
Food-related myths				
Drinking from plastic bottles	88 (15.7)	1,023 (25.2)	0.62 (0.48 to 0.80)	<.001
Eating food containing artificial sweeteners (eg, saccharine)	94 (16.8)	811 (20.0)	0.85 (0.66 to 1.09)	.20
Eating genetically modified food (eg, hybrid vegetables)	108 (19.3)	569 (14.0)	1.37 (1.07 to 1.74)	.013
Eating food containing additives	84 (15.0)	372 (9.2)	2.01 (1.52 to 2.66)	<.001
Using microwave ovens	155 (27.7)	813 (20.0)	1.49 (1.20 to 1.85)	<.001
Eating burnt food (eg, bread or barbeque)	176 (31.4)	981 (24.1)	1.76 (1.43 to 2.16)	<.001
Food-unrelated myths				
Using aerosol containers	208 (37.1)	1,850 (45.5)	0.78 (0.64 to 0.94)	.010
Using mobile phones	193 (34.5)	1,532 (37.7)	1.00 (0.82 to 1.22)	.97
Using cleaning products	184 (32.9)	1,624 (40.0)	0.79 (0.65 to 0.97)	.023
Living near power lines	172 (30.7)	1,368 (33.7)	0.86 (0.70 to 1.05)	.15
Feeling stressed	179 (32.0)	1,270 (31.3)	1.07 (0.87 to 1.31)	.53
Having a physical trauma	396 (70.7)	2,356 (58.0)	1.92 (1.57 to 2.35)	<.001
Exposure to electromagnetic frequencies (eg, Wi-Fi and Radio/TV frequencies)	116 (20.7)	1,372 (33.8)	0.58 (0.46 to 0.72)	<.001

Abbreviation: OR, odds ratio.

^a^ Adjusted for age group, sex, educational level, occupation, monthly income, place of residency, marital status, the presence of a chronic disease, knowing someone with cancer, and data collection site.

Having a physical trauma was the most recognized food-unrelated myth in both nonvegetarians (n = 2,356, 58.0%) and vegetarians (n = 396, 70.7%). By contrast, the least recognized food-unrelated myth was feeling stressed (n = 1,270, 31.3%) in the nonvegetarian group and exposure to electromagnetic frequencies (n = 116, 20.7%) in the vegetarian group.

Vegetarians were more likely than nonvegetarians to recognize five of 13 CRC risk causation, namely, eating genetically modified food (OR, 1.37 [95% CI, 1.07 to 1.74]), eating food containing additives (OR, 2.01 [95% CI, 1.52 to 2.66]), using microwave ovens (OR, 1.49 [95% CI, 1.20 to 1.85]), eating burnt food (OR, 1.76 [95% CI, 1.43 to 2.16]), and having a physical trauma (OR, 1.92 [95% CI, 1.57 to 2.35]). On the other hand, vegetarians were less likely to recognize four other CRC causation myths, namely, drinking from plastic bottles (OR, 0.62 [95% CI, 0.48 to 0.80]), using aerosol containers (OR, 0.78 [95% CI, 0.64 to 0.94]), using cleaning products (OR, 0.79 [95% CI, 0.65 to 0.97]), and exposure to electromagnetic frequencies (OR, 0.58 [95% CI, 0.46 to 0.72]).

### Good Awareness and Its Associated Factors

A total of 1,698 nonvegetarians (41.8%) demonstrated good awareness of CRC signs/symptoms, whereas 151 vegetarians (27.0%) showed similar awareness (Table 5). Multivariable logistic regression analysis revealed that being a vegetarian was associated with a lower likelihood of displaying good CRC symptom awareness (OR, 0.59 [95% CI, 0.48 to 0.72]; Table 6).

**TABLE 5:** Awareness Level of Colorectal Cancer Symptoms, Risk Factors, and Causation Myths

Domain	Vegetarian (n = 560), No. (%)	Nonvegetarian (n = 4,063), No. (%)	*P*
Colorectal cancer symptoms			<.001
Poor	156 (27.9)	677 (16.7)	
Fair	253 (45.2)	1,688 (41.5)	
Good	151 (27.0)	1,698 (41.8)	
Colorectal cancer risk factors			.36
Poor	50 (8.9)	350 (8.6)	
Fair	273 (48.8)	2,110 (51.9)	
Good	237 (42.3)	1,603 (39.5)	
Colorectal cancer causation myths			.13
Poor	353 (63.0)	2,514 (61.9)	
Fair	190 (33.9)	1,347 (33.2)	
Good	17 (3.0)	202 (5.0)	

**TABLE 6:** Univariable and Multivariable Logistic Regression Analyses Examining the Association of Being a Vegetarian With Displaying Good Awareness of Each of Colorectal Cancer Symptoms, Risk Factors, and Causation Myths

Domain	Univariable Analysis		Multivariable Analysis	
	Crude OR (95% CI)	*P*	Adjusted OR^a^ (95% CI)	*P*
Colorectal cancer symptoms	0.51 (0.42 to 0.63)	<.001	0.59 (0.48 to 0.72)	<.001
Colorectal cancer risk factors	1.13 (0.94 to 1.35)	.19	0.93 (0.77 to 1.12)	.45
Colorectal cancer causation myths	0.60 (0.36 to 0.99)	.045	0.90 (0.53 to 1.52)	.69

Abbreviation: OR, odds ratio.

^a^ Adjusted for age group, sex, educational level, occupation, monthly income, place of residency, marital status, the presence of a chronic disease, knowing someone with cancer, and data collection site.

The majority in both the vegetarian and nonvegetarian groups displayed fair awareness levels of CRC risk factors (48.8% and 51.9%, respectively). By contrast, the awareness level of CRC causation myths was poor in both groups, with 63% of vegetarians (n = 353) and 61.9% of nonvegetarians (n = 2,514) demonstrating poor awareness. However, there were no significant associations between being a vegetarian and the awareness levels of CRC risk factors and causation myths.

## DISCUSSION

The findings of this study revealed that, overall, awareness of CRC signs/symptoms was inadequate in both groups. In addition, most participants in both groups displayed fair awareness of CRC risk factors. Moreover, public awareness of mythical causes of CRC was similarly poor in both vegetarians and nonvegetarians. This shared knowledge deficit highlights the need for accurate information dissemination as it can directly influence individuals’ health behaviors. Studies have shown that individuals who perceive cancer prevention as beyond their control are less likely to engage in preventive measures.^30^ Therefore, addressing these prevalent myths through targeted awareness campaigns becomes crucial in promoting informed and proactive health behaviors.

Vegetarians exhibited significantly lower awareness levels of CRC signs/symptoms, with only 27.0% demonstrating good awareness, compared with nonvegetarians (41.8%). Moreover, vegetarians were significantly less likely to recognize most CRC signs/symptoms. The most recognized sign/symptom among both groups was lump in the abdomen. These results align with a previous study conducted in Palestine that assessed public awareness levels of CRC signs and symptoms. The study also explored factors associated with having good CRC symptom awareness in the general Palestinian population, where following a vegetarian diet was associated with lower odds of having good awareness.^7^ This emphasizes the persistent challenge of low CRC awareness in this population and the imperative need for CRC awareness initiatives in Palestine, especially in the absence of an existing screening program.^31^

Both groups identified lack of physical activity as a prominent modifiable risk factor. This consensus could be attributed to various campaigns promoting physical activity in Palestine.^8^ Interestingly, vegetarians demonstrated a lower tendency to recognize red meat consumption as a modifiable risk factor for CRC, which could be attributed to their dietary preferences, characterized by limited to no red meat consumption. Instead, as supported by our study findings, vegetarians seemed to prioritize other risk factors, such as physical activity, which they may perceive as more relevant to their lifestyle choices. Another plausible explanation may relate to their lower income levels. Our study indicated that only 40% of vegetarians had a monthly income exceeding the minimum wage. This limited income could result in reduced access to health care and medical education, which in turn might have contributed to lower awareness levels. In addition, it is worth considering that the economic constraints might have played a role in their decision to adopt a vegetarian diet as they might have been unable to afford meat products. Consequently, awareness campaigns should be tailored to address these specific knowledge gaps within the targeted population, especially given the complex interplay between dietary choices and awareness levels.

Our study identified variations in the attribution of mythical causes of CRC among participants. Having a physical trauma was the most frequently reported mythical cause of CRC in both groups although this misconception was more pronounced among vegetarians. Notably, myths like drinking from plastic bottles and eating burnt food received higher endorsement as potential causes, whereas eating food additives and exposure to electromagnetic frequencies were less commonly linked to CRC. These findings contrast with a study in Western Australia, which reported a high level of endorsement for unestablished cancer risk factors, including food additives and high voltage power lines.^32^ However, other studies have observed diminished levels of endorsement for these unestablished risk factors.^33,34^ Another study in the United Kingdom assessing the prevalence of beliefs about actual and mythical causes of cancer reported that approximately one third of the participants associate cancer risk with factors such as stress, genetically modified food, food additives, and nonionizing electromagnetic frequencies.^26^ These findings highlight the importance of continuous monitoring of public risk perceptions and the need to enhance awareness regarding misconceptions surrounding CRC causation.

The increasing endorsement of mythical causes of cancer observed in recent years could potentially be attributed to shifts in individuals’ patterns of accessing and consuming news and information.^35^ A systematic review on social media and CRC concluded that individuals diagnosed with CRC and their family members are progressively using social media platforms.^36^ A significant proportion of these platforms lack authoritative validation, and the information they disseminate is frequently anecdotal and devoid of scientific substantiation.^36^ Nevertheless, social media offers several advantages over traditional information sources as it can reach a broader population of patients with CRC and individuals at risk. Therefore, harnessing the potential of social media to dispel common misconceptions about CRC in Palestine could be a valuable strategy for improving public awareness and promoting evidence-based knowledge.

The results of the study highlight the importance of developing tailored awareness campaigns as a promising approach to address the knowledge gaps observed among vegetarians. In particular, it emphasizes the need for in-person and virtual campaigns to improve the low awareness of CRC signs/symptoms within this group. These campaigns should be thoughtfully designed to accommodate the unique dietary preferences and lifestyles of this group, with a specific emphasis on CRC risk factors relevant to their choices. Furthermore, efforts should be made to address specific areas of awareness deficiency, such as the lack of recognition of diabetes as a risk factor for CRC.

There are certain limitations that should be considered while interpreting the results. First, the use of convenience sampling may not create a fully representative sample of the Palestinian population, thus limiting the generalizability of the study findings. Nonetheless, the study’s large sample size, high response rate, and data collection from diverse regions across Palestine might have helped to mitigate this concern. Another limitation is the exclusion of individuals visiting oncology departments and those with medical backgrounds. While this might have reduced the number of participants with presumed good CRC awareness, it was done to maintain the study’s primary focus on assessing CRC in the groups of interest. The study also assessed the perceived knowledge of participants, rather than individuals with actual CRC symptoms. Finally, the definition of a vegetarian diet was self-reported, and this definition could be different in other regions.
